# Meta-analysis of Supply Chain Disruption Research

**DOI:** 10.1007/s43069-021-00118-4

**Published:** 2022-02-02

**Authors:** Lydia Novoszel, Tina Wakolbinger

**Affiliations:** Wirtschaftsuniversität Wien: Wirtschaftsuniversitat Wien, Vienna, Austria

**Keywords:** Meta-literature review, Disruption, Disaster, Pandemic, Supply chain, Humanitarian, Sustainability, Performance

## Abstract

The purpose of this chapter is to provide insights into literature on supply chain disruption research with a specific focus on future research opportunities. A structured meta-literature review approach covering 93 literature reviews was chosen. Quantitative and qualitative content analysis and bibliographic network analysis are applied to highlight trends and research gaps. The meta-analysis shows the current and past academic discourse on supply chain disruptions. Furthermore, this research establishes a research framework and highlights future research opportunities. The research points to research topics that should be addressed in the future. The paper provides a holistic understanding of literature on supply chain disruptions in the commercial and humanitarian context.

## Introduction

Supply chain disruptions result from unforeseen or unplanned events that interrupt the regular flow of goods within a supply chain [1–3]. During the COVID-19 pandemic, supply, demand and distribution disruptions are happening simultaneously [4, 5]. First surveys among practitioners indicate strong implications of the crisis for commercial and humanitarian supply chains. Seventy-three percent of commercial supply chains in the USA experienced changes in their supply and 75% in their production and distribution [6]. Almost all humanitarian organizations applied changes to their operations and 93% got impacted due to actions by authorities [7]. Forty percent recognized increased needs from beneficiaries [8].

The global COVID-19 pandemic also sparked and accelerated research on supply chain disruptions. In order to understand the current academic discourse, a meta-review of existing literature reviews is chosen. Based on the analyzed literature reviews, a research framework is developed and future research opportunities are identified.

The paper is structured as follows: The first section describes the research method and study design. The bibliographic information is part of the second section, followed by a keyword analysis. Next, the research framework and research opportunities are presented. The conclusion section summarizes the main insights of the chapter.

## Research Method and Study Design

This paper uses a systematic literature review [9] to investigate literature reviews of disruption research. The goal is to synthesize research findings in a systematic, transparent and reproducible way [9]. The main stages according to Tranfield et al. [10] are as follows: planning the review, conducting a review and reporting & dissemination. Levitt [11] describes how to conduct a qualitative meta-analysis based on systematically selected primary literature. The primary findings are labeled by creating categories based on commonalities and distinctions. These labels and their meaning examine the relationships to central insights of the investigated field. This paper applies the PRISMA (Preferred Reporting Items for Systematic reviews and Meta-Analyses) approach outlined by Moher et al. [12] to document the research approach. The steps, which lead to the final set of relevant papers that build the sample data for this review, are captured in Fig. 2.

The research questions have been formulated based on Denyer and Tranfield [13] using CIMO (context, intervention, method, outcome) logic.

Context (C)

RQ1: What is the supply chain context of the review (commercial, humanitarian or public supply chain)

Interventions (I)

RQ2: Which sources of disruptions are identified?

RQ3: Which stages of the supply chain are disrupted?

Methods (M)

RQ4: Are applications of quantitative tools/methods investigated?

Outcomes (O)

RQ5: Is the impact of disruptions on performance considered, if yes how?

RQ6: Which research gaps and further research areas are suggested?

**Fig. 1:**
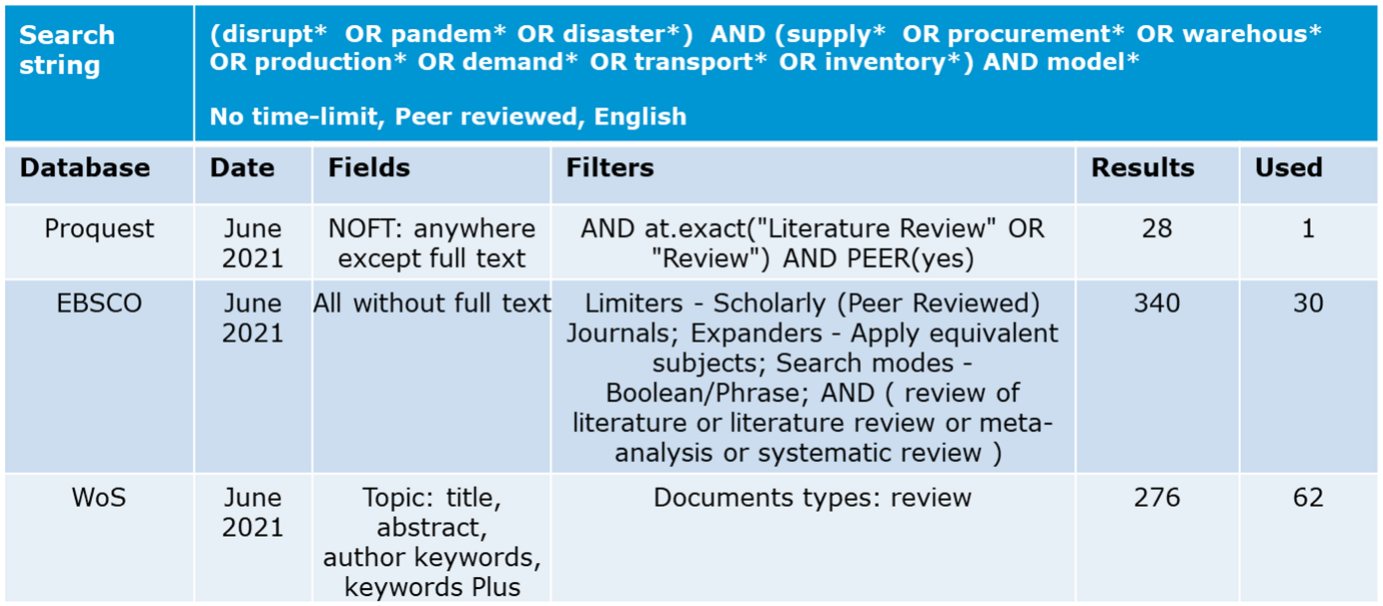
Search protocol. Databases Proquest (www.proquest.com/), EBSCO (www.ebsco.com), and Web of Science (WoS, www.webofscience.com/wos/woscc/) were chosen for a wide array of publishers of journals and led to initial results between 28 and 340 papers

The outcome of the database search conducted in June 2021 is outlined in Fig. 1. Based on the research questions, the search string for the analysis consists of four elements: first, keywords that are linked to disruptions as such (for example disasters, since this term is used in the humanitarian sphere) and pandemic due to the recent COVID-19 challenges. The next components are linked to supply chains and their functional areas, such as supply, procurement, production and transportation. In order to identify literature review publications, the respective filter and identifier was used in the databases. Only peer-reviewed papers in English were searched. Figure 1 shows the details of the used search string, databases, fields and filters.

**Fig. 2:**
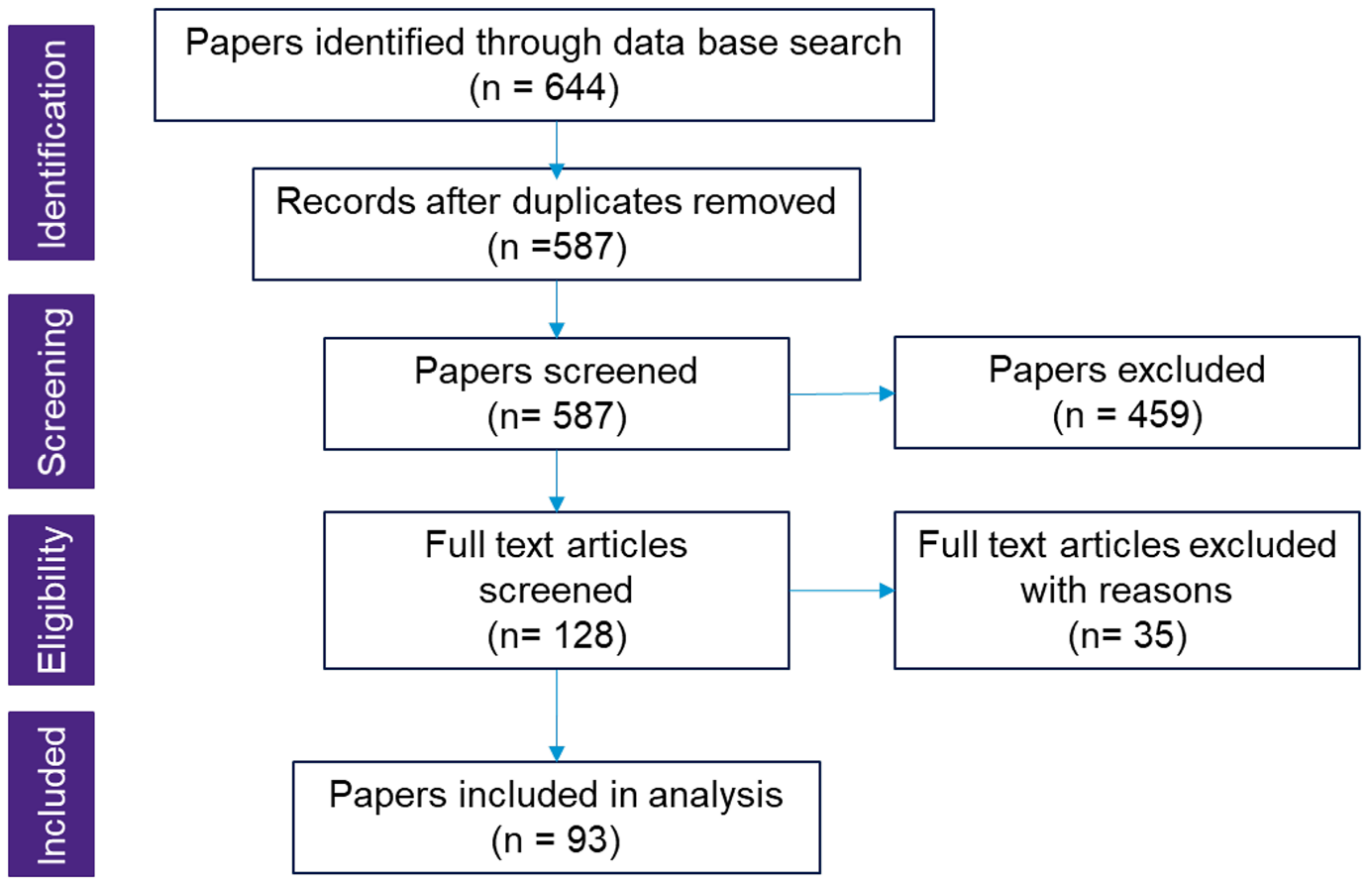
Review protocol based on Moher et al. [12]

The systematic review was conducted following the PRISMA flow logic. For illustration of the steps and outcomes, refer to Fig. 2. The identification step comprises the 644 papers identified through the database search. Removing duplicates (57 papers) led to 587 papers that were reviewed based on title and abstract. Four main exclusion criteria were applied during the screening phase: the research method of the paper (not a literature review—56 publications), medical literature (298 articles), focus on disruptive technology (such as AI and block chain) rather than on supply chain disruptions (36 papers) and the missing link to supply chain (disruption) overall (69 papers). During the eligibility phase, 128 articles were reviewed in detail by reading the full text. Thirty-five further papers were excluded due to the criteria established in the screening phase. Finally, in total 93 literature review papers were included in the analysis of the supply chain disruption research.

### Bibliographic Analysis

In order to understand the structure of the investigated papers and the publication dynamic, a bibliographic and network analysis of the identified 93 papers was conducted. This information is relevant for quantitatively organizing available knowledge within a scientific discipline [14, 15]. The selected articles were published between 2006 and June 2021. Since there was no time-constraint used in the search criteria, 2006 marks the first year of a literature review published on supply chain disruption research. The paper from Altay and Green [16] investigates publications from 1980 on, which references early publications from Sampson and Smith [17] and Sheffi et al. [18]. Between 2006 and 2018, 0 to 9 review papers were published yearly. From 2019 publications increased with 21 published review articles in 2020. The database research was conducted until June 2021, with the last paper included from Sharma et al. [19]. The most citied paper is by Tang [20], a review on “perspectives in supply chain risk management.” It identifies four basic approaches for managing supply chain risks: supply, demand, information and product management.

**Fig. 3:**
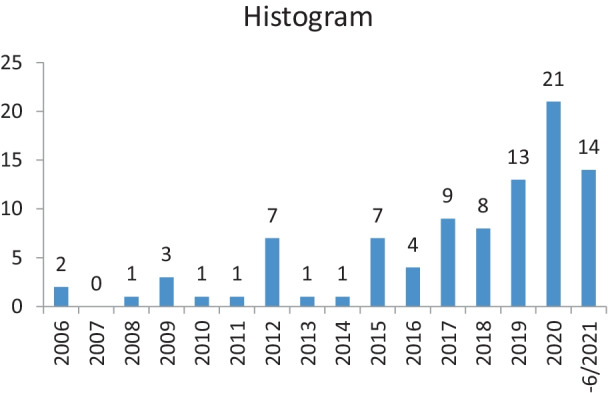
Yearly distribution of publications of literature reviews

Let us highlight that the scope of the review is investigating published literature reviews. The array of articles linked to supply chain disruptions and especially pandemic and COVID-19 is even wider. Papers that apply methodology other than a literature review are not considered in our analysis (Fig. 3).

**Fig. 4:**
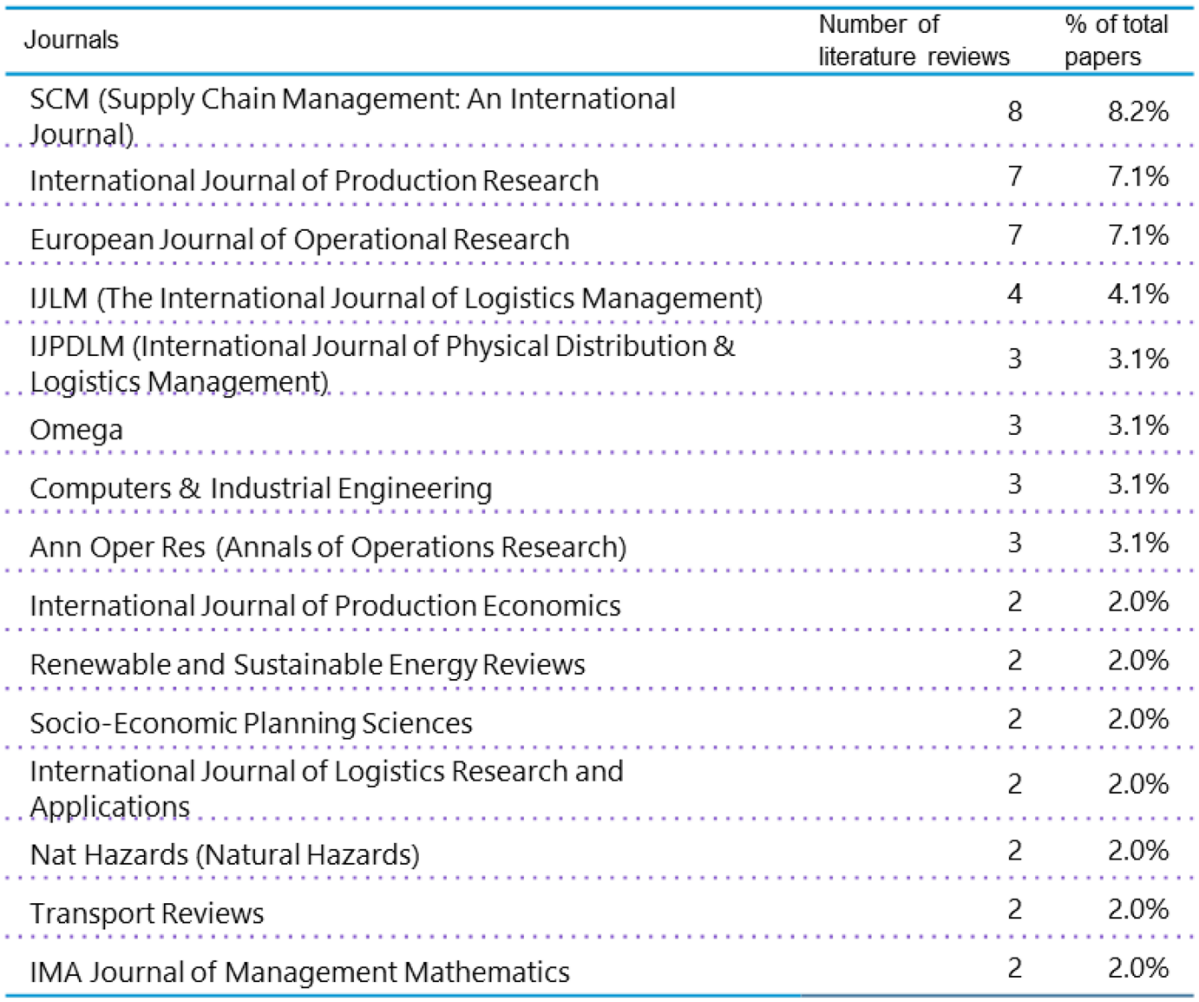
Journals with more than 1 literature review dealing with supply chain disruptions

In total, 93 literature reviews were published in 56 different journals. Figure 4 shows the list of journals with more than one publication as part of this analysis. The wide array of subjects covered by the journals is an indication for the cross-functionality of supply chain disruption research. The top three journals with respect to the number of literature reviews are SCM (Supply Chain Management: An International Journal) (8 publications), the International Journal of Production Research (7 publications), and European Journal of Operational Research (7 publications).

### Keyword Analysis

This section focuses on the content of the literature reviews under investigation in this study. A quantitative analysis of (key) words provides insights on the covered topics and used terms.

**Fig. 5:**
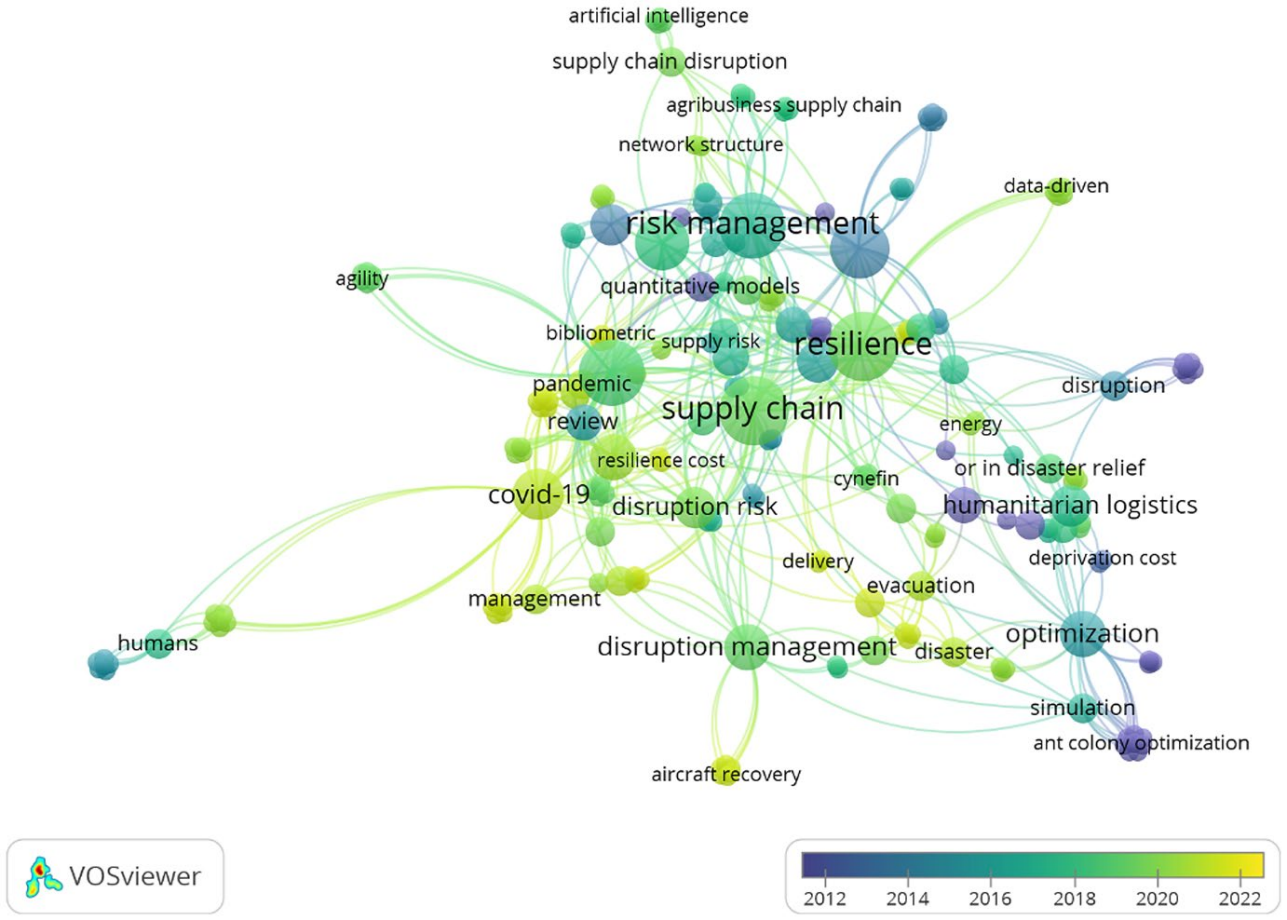
Author picked keyword overview

As baseline for the content analysis, a review of the author picked key words was conducted and visualized with the VOSviewer application [21], (https://www.vosviewer.com/). Figure 5 highlights the key word usage over time, where resilience and COVID-19 appear more recently (around 2020), whereas risk management seems to have been used earlier on (around 2016).

**Fig. 6:**
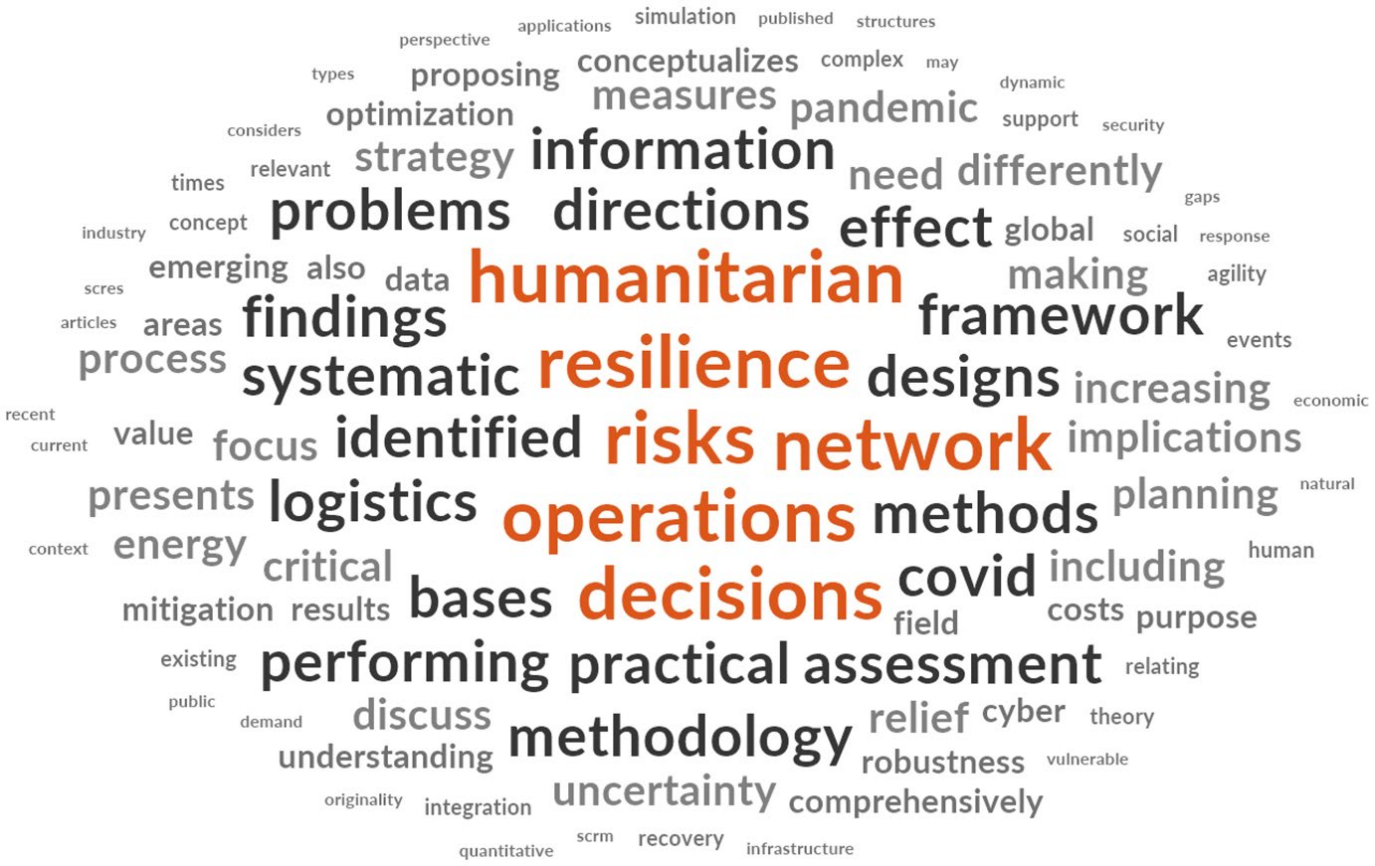
Word cloud of author picked words in titles, keywords, and abstracts

In order to visualize words used in the title, author picked keywords and abstracts, a word cloud (see Fig. 6) was constructed to get additional insights on the key terms from the literature reviews.

Looking at the word count of this dataset, while eliminating search terms and fill words, the top 5 (out of 250) words used are: risk (count of 206, 1.90% weighted percentage), resilience (190, 1.74%), operations (103, 0.95%), network (102, 0.94%), and humanitarian (74, 0.64%). We would like to emphasize, that these words are the result of the search, since they were not included in the search string (see study design). This indicates that risk, resilience, and humanitarian are closely linked to supply chain disruption research.

### Research Framework

**Fig. 7:**
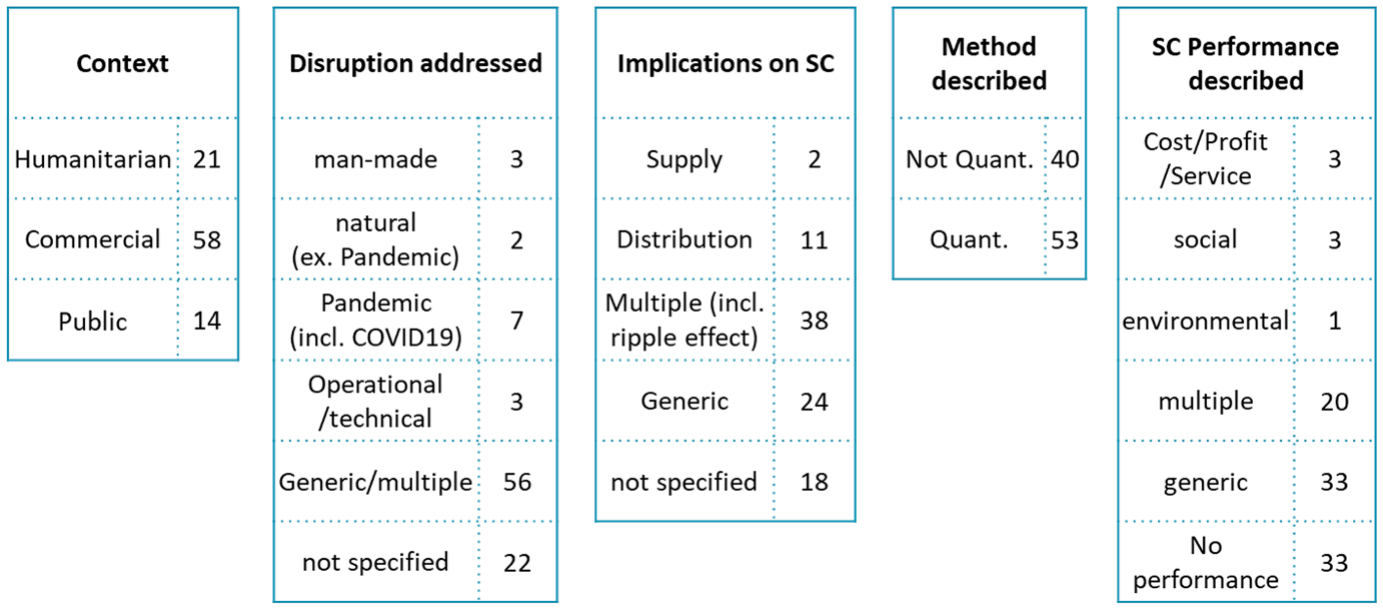
Supply chain disruption research framework with allocated literature reviews

In order to structure supply chain disruption research, we propose the following framework (see Fig. 7). It was developed based on the research questions following the CIMO logic from Denyer and Tranfield [13]. Different supply chain purposes (commercial, humanitarian, public) build the context (C). Disruptions mark interventions (I) to supply chains. On the one hand, disturbances can have natural, man-made and operational causes. On the other side, the implications on supply chains can happen on supply, demand and distribution/infrastructure side. Quantitative and qualitative research methods (mechanisms, M) can be applied to investigate supply can disruption research. The outcome (O) of disturbances on supply chains can be identified by supply chain performance and its different dimensions (such as monetary- and sustainability targets covering also ecological and societal ambitions).

To structure the investigated literature, the different dimensions were clustered according to their appearance and each paper was allocated once. The category “generic” (used in the following dimensions: disruption addressed, implications on supply chain and supply chain performance described) summarizes papers that mention the term, but do not elaborate on specifics. “Not specified” (and analogical “no performance”) indicates that the dimension (disruption, implication, performance) is not covered in the review. We now address each one of the dimensions of the framework in detail.

#### Context

**Fig. 8:**
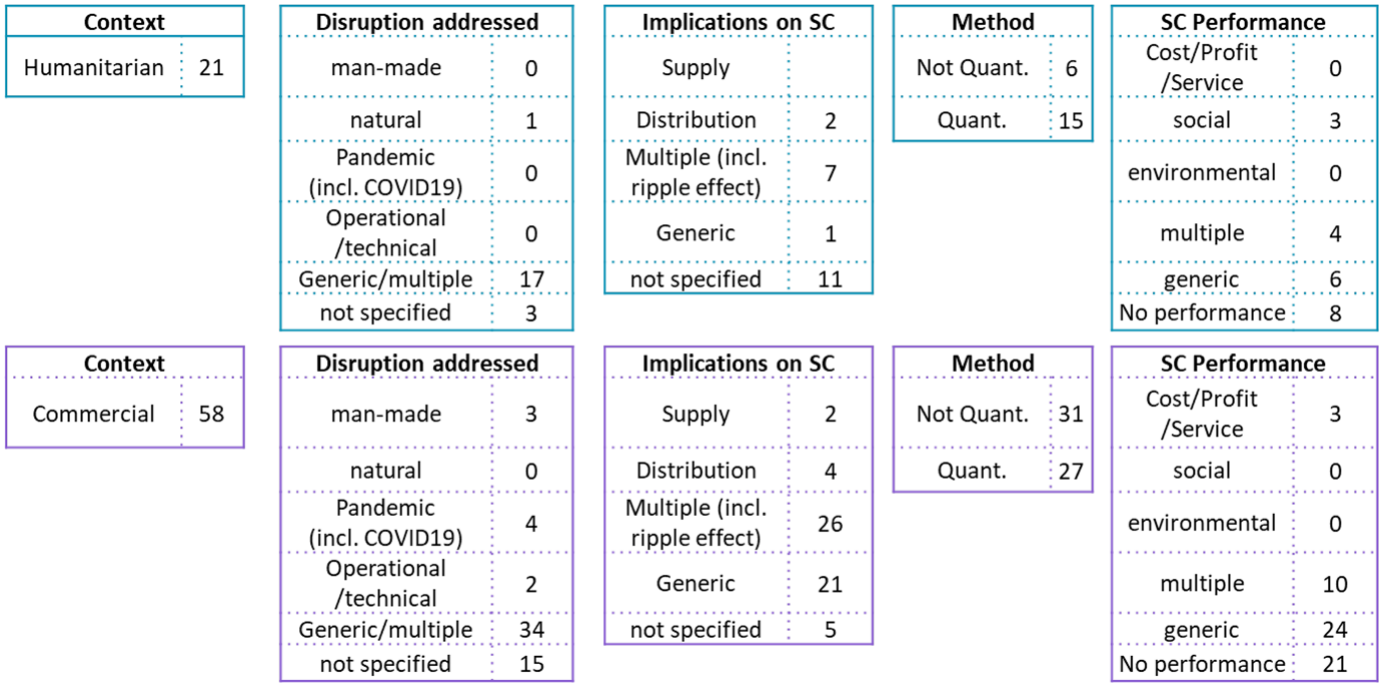
Supply chain disruption research framework comparing humanitarian versus commercial approaches

Differences and similarities between humanitarian and commercial supply chains have been widely researched (e.g., [22–26]. In this paper, we deliberately added keywords, such as “disaster” to incorporate the humanitarian perspective into the meta-review of supply chain disruption research. This allows us to compare insights from the commercial and humanitarian sector. Figure 8 illustrates the suggested framework for supply chain disruption research and allocates the investigated literature reviews based on the humanitarian and commercial context.

The majority (58) of the investigated literature reviews has a focus on commercial supply chains. These papers mainly address generic (multiple) disruptions, but also highlight (COVID-19) pandemics. Multiple implications on supply chains (such as supply, demand, operations and propagation) are investigated. The “not specific” assignment is higher in the area of disruption sources (such as man-made versus natural disaster) than in the supply chain dimension. This might indicate that the external reason for the disruptions is not as important as the concrete supply chain disruption, which triggers the recovery activities. Both quantitative as well as non-quantitative perspectives are considered in the literature reviews. When considering supply chain performance, the focus is on generic or no performance investigations.

Literature reviews in the context of humanitarian supply chains address generic and multiple disruptions and do not put a specific emphasis on supply chain implications. The methods investigated are mainly quantitative research methods. Supply chain performance seems to be mostly not considered, or rather generic. The set of literature reviews that form the basis for this meta-analysis do not include a literature review with a specific focus on pandemics. However, Queiroz et al. [27] indicate that humanitarian literature has extensively studied epidemic impacts and identifies a research gap in understanding pandemic impacts in commercial supply chains.

The main difference between humanitarian and commercial context seems to be, that the first one focuses on the external disruptions whereas the later one puts an emphasis on the supply chain implications. Moreover, the implications of supply chain disruptions on performance seem to be more researched within commercial supply chains.

#### Disruptions Addressed

Disruptions can have natural or man-made causes. Natural disasters refer to events, such as floods, earthquakes, hurricanes or pandemics (e.g., [28, 29]. Man-made root causes can link to wars, terrorist- or cyber-attacks or mistakes that lead to operational interruptions. Van Wassenhove [30] additionally distinguishes between slow- and sudden onset disasters. For the purpose of this study, we categorize the literature reviews into generic/multiple, operational/technical, man-made, natural (excluding pandemics) and pandemic crisis. Due to the ongoing global COVID-19 pandemic, it seems of interest to specifically indicate papers with a pandemic focus. Literature reviews, which do not address this kind of root causes, are coded as “not specified” (22).

The majority of the papers can be classified in the category generic/multiple (e.g., [31–33]. The following literature reviews have a direct link to the COVID-19 pandemic: Black and Glaser-Segura [34], Cordeiro et al. [35], Davahli et al. [36], Gkiotsalitis and Cats [37], Golan et al. [38] and Singh et al. [39]. Lusby et al. [40], Colicchia et al. [41] and Christersson and Rothe [42] investigate operational disruptions. Natural disasters are specifically addressed by Emodi et al. [43] and Seaberg et al. [44]. Cyber-attacks are one example of man-made disasters, which are researched by Parn and Edwards [45] as well as Ghadge et al. [46].

In summary, overall generic and multiple disruptions are investigated; also, the COVID-19 pandemic is getting attention. Some literature reviews do not indicate root causes for disruptions.

#### Implications on Supply Chains

Supply chain disruptions are defined as unexpected and unforeseen events or circumstances that disturb the regular flow of goods and materials along the value chain [1–3]. This can happen due to shortage of supply parts, disturbances during operations and distribution or changes from a demand perspective [5, 47, 48]. Suppliers might be impacted in their ability to produce due to lack of raw materials, funds, trained labor or less efficient production processes caused by natural or man-made disasters (examples amid COVID-19 are Attinasi et al. [49], Keshner [50] or Souza [48]. Distribution capacities can be affected due to changes in border controls, availability of transportation infrastructure (e.g., roads, ports, canals, belly freight cargo-space) and available labor capacities. Gossler et al. [51] have recently highlighted how transportation activities can improve the success of humanitarian operations. Earlier publications (e.g., [52] provide more comprehensive research on the relevance of transportation networks. On the demand side, changing customer needs lead to disruptions along the value chain [53]. These shifts in market requirements can be triggered because of psychological phenomena (such as hoarding or behavior changes) or additional application needs. The disturbances in various nodes of the supply chain can spread across the connected value chain network. The propagation of disruptions are described by the ripple effect [54, 55] and the bullwhip effect [56]. These kinds of supply chain disruptions are also investigated during the ongoing COVID-19 pandemic [4, 5].

The majority of the literature reviews addresses multiple supply chain disruptions. For example, disruptions in the supply and demand stage are considered by Singh et al. [39] and Manuj and Mentzer [57]. Hosseini and Ivanov [54], Hosseini et al. [58] and Llaguno et al. [55] investigate the ripple effect. Multiple papers do not explicitly investigate various possible supply chain disruptions, but rather look at general vulnerabilities or variations in value chains (e.g., [16, 38, 59–61]. Some papers do not consider a specific supply chain disruption (for example [62–65]. Other reviews can be linked to distribution or infrastructure disturbances (e.g., [36, 40, 66]. Two papers solely highlight supply disruptions in commercial supply chains [67, 68]

#### Methods Described

The search string for the study includes the key word “model.” This was chosen in order to put an emphasis on models described or used in the literature reviews. The majority of the papers (53) focuses on investigating quantitative models.

For example, Caunhye et al. [69] investigate optimization models in emergency logistics. The literature is structured based on data type (stochastic or deterministic), levels (single-level or bi-level), and (single or multilevel) objectives. Altay and Green [16] apply the structure of Denizel et al. [70] to characterize different disaster operations management activities according to the disaster management cycle: model development, theory development, and application (tool) development. In 2019, Hosseini et al. introduce a structured analysis and recommendations concerning which quantitative methods can be used at different levels of capacity resilience. In their 2020 paper, Hosseini and Ivanov specifically focus on Bayesian networks for supply chain risk, resilience, and ripple effect analysis. Within the public context, for example, Bešinović [71] reviews methods to estimate resilience of railway transport systems, such as mathematical optimization, topological, simulation, optimization, and data-driven approaches.

The other (40) literature reviews do not look into quantitative methods. They focus more on descriptive topics to understand supply chain disruptions and link them to theories, such as supply chain resilience (e.g., [34, 63, 72–77], supply chain risk management (e.g., [41, 59, 62, 78, 79], and mitigation actions for recovery (e.g., [39, 80, 81].

#### Supply Chain Performance

**Fig. 9:**
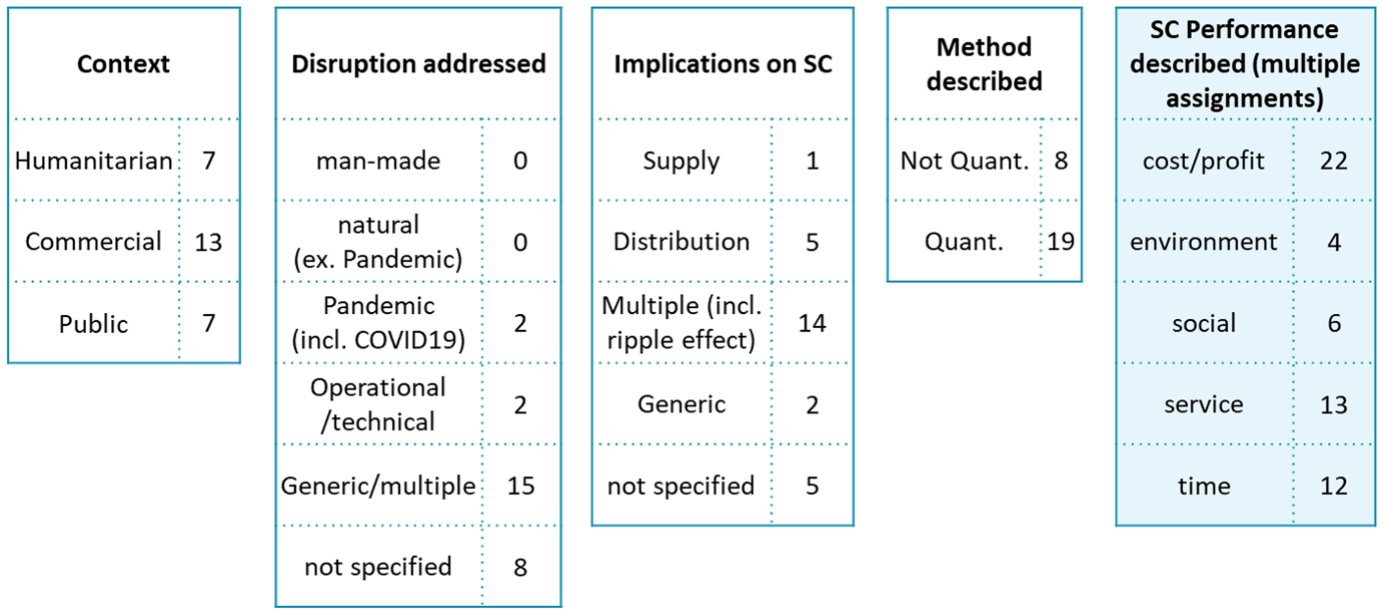
Supply chain disruption research framework focusing on supply chain performance

This section is based on the 27 literature reviews that discuss performance implications due to supply chain disruptions. Excluded are 66 literature reviews, which have no (33) or very generic (33) performance considerations. Supply chain performance plays a role in evaluating supply chain activities and impacts strategic, tactical and operational planning [82]. It can have multiple dimensions. Monetary indicators, such as cost or profit, can describe the economic efficiency of supply chain activities [83, 84]. Service levels indicate if, for example, time and qualitative expectations are met and thus can stipulate customer satisfaction [83, 84]. A more holistic view is attempted with sustainability and triple-bottom-line approaches (for example see Kleindorfer et al. [85] or Elkington [86], which also incorporate an environmental and social perspective. During supply chain disruptions, time to react or recover are metrics, which are relevant to understand the duration of the impact on the supply chain, until flow of goods/information/funds is re-established. Figure 9 summarizes which elements (multiple assignments possible) the 27 identified papers cover and how it links back to other dimensions of the framework.

The most mentioned performance dimension is linked to cost or profit [16, 37, 40, 42, 58, 59, 63, 64, 67, 69, 87–97] across all contexts (humanitarian, commercial and public). For example, Lusby et al. [40] investigate minimizing costs in disaster relief distribution models. Heckmann et al. [87] take monetary figures (profit-, cost-, or cash-flow-oriented) under consideration when describing supply chain risks as the deviation of the affected objective.

Service level and demand implications are described by 13 literature reviews [58, 59, 63, 64, 69, 87–89, 91, 97–100]. Shen and Li [89] consider service and demand as part of the supply chain profit function.

Time is mostly considered within public supply chains (such as vaccines, energy and transport infrastructure). Ahmadi et al. [92] describe quantification of energy system resilience, being a function of time. Within the commercial context, for example, Hosseini et al. [58] include recovery time as one element of the supply chain resilience objective function. An example of time consideration within the humanitarian supply chain perspective is the literature review by La Torre et al. [97] that looks into minimum total response time in disaster relief distribution models.

Environmental and social aspects are least considered. Commercial perspectives seem to more often consider environmental targets (for example [42, 93]. Whereas, humanitarian supply chain research looks more often into social and humanitarian implications [64, 95, 96]. Gajanayake et al. [101] elaborate on direct versus indirect impacts and tangible (mainly economic) and intangible (mainly social and environmental) measurements (referring to [102].

Disaster-related impacts might also have positive side effects in both commercial as well as humanitarian contexts. The COVID-19 pandemic shows, how a global pandemic can trigger product innovation (e.g., vaccine development, refer to, e.g., [103]), repurposing of resources (e.g., automakers producing ventilators, see [104]) and acceleration of trends (e.g., digitalization, see [105]). Gains for communities can result in form of aid flow, increased employment or enhancement of the natural environment [101].

In summary, literature reviews in this study seem to focus on monetary targets, followed by considerations on service levels and time, when analyzing implications of supply chain disruptions.

### Synthesis of Identified Research Opportunities

The following section provides a synthesis of future research opportunities provided by the 21 papers in the humanitarian context and 58 articles from the commercial perspective. The time-range of these publications is from 2006 up to June 2021. The literature reviews capture trends and academic possibilities over a time span of 15 years, some of which might have been addressed over the course of the time.

#### Definition and Characteristics of Resilience

Various authors ask for a clear, holistic definition and consideration of supply chain resilience [38, 63, 76, 90, 106]. Ponomarov and Holcomb [76] also highlight the clear definition of the phenomenon of resilience and the relationship between supply chain capabilities and supply chain resilience. Hohenstein et al. [63] emphasize a strong need for an overarching supply chain resilience definition and a clear terminology for resilience building blocks. Also in most recent publications, a broad up-dated definition of supply chain resilience is requested, with additional details, such as the consideration of different types of disruptions or risks [38, 106] and linking the “R” of resilience concept to triple-A [107] supply chain [72].

Humanitarian literature reviews highlight the implications of supply chain design on agility and the circumstances of the different disaster management phases [108]. Oloruntoba and Kovács [108] also express missing clarity over pursuing agility, resilience, risk reduction or sustainability criteria in long-term (permanent) humanitarian aid supply chains. This links to questions raised concerning the definition of resilience, its implications on performance, and trade-offs with other paradigms. However, future research will show if choices need to be made, or if different concepts can be encompassed together with sustainability targets in mind.

#### Measurement of Resilience, Impact of Supply Chain Disruptions, and Mitigation Actions on Supply Chain Performance

Along with the quest for a cohesive resilience definition comes the question regarding measurement of supply chain resilience [57, 61, 63, 73, 75, 76, 80, 88, 90, 91, 99, 109]. Hosseini et al. [58] mention Ojha et al. [110], who developed a metric to quantify resilience as a measure of service loss in the aftermath of disruption. Resilience can be described as a capability [99] to combat unforeseen supply chain disruptions due to various causes. Its attempt is to enable supply chains to recover from disturbances, with as little negative effect on performance [55] as possible. Hence, it is important to understand the relationship between resilience strategies (and mechanisms) and performance [63] as well as indicators such as recovery time or speed [57]. New key performance indicators can integrate operability objectives (e.g., resilience, stability, robustness [88]). Moreover, they could define supply chain objectives for different nodes [75, 81] to incorporate into supply chain design decisions. Visibility [81] and vulnerability [19] parameters [68] can be included into a performance management system for supply chain disruption management. Looking into varying demand patterns and studying the effect that disruptions in demand (and sales) have on supply chain performance are mentioned by Llaguno et al. [55]. It could be of interest to analyze different strategies [93, 99], policies and measures to address disruptions and to recover from them [55]. Another aspect could be investigating the timing of activities [57], the associated costs [93, 111], and how they link to performance outcomes and efficiency [93]**.** Opportunities for competitive advantages (or priorities, see e.g. [99]) under disruptions without compromising performance in everyday situations could also be researched.

#### Consideration of Sustainability (Triple-Bottom-Line) and Circular Supply Chains

The concepts of sustainability [85] and triple-bottom-line [86] enhance the monetary view of supply chain outcomes by considering economical, ecological, and social factors [112]. The impacts of disruptions on sustainability [33, 55, 93, 113] and circular supply chains [93] are investigated. Moreover, sustainability could serve as a potential solution for more resilient responses of supply chains towards disruptions [58, 93]. It could be worthwhile to investigate which impact the proactive and reactive measures to combat disruptions have on environmental, economic and social criteria [55] and how social welfare varies in the presence of disruptions [89]. Combinations of concepts and paradigms like circular supply chains, sustainability and the ripple effect [114], as well as studying the interface between green and resilient supply chains [58] can enhance the understanding of value chain behavior amid disturbances. There is a lack of research on direct and indirect environmental and social impacts of disasters [101]. The reason could be that a debate exists on how to measure social and environmental impacts. A more holistic view on wider environmental impacts, besides carbon emissions, is encouraged by Gajanayake et al. [101], which might be beneficial in the future. Within the humanitarian context, reverse logistics flows for recovery such as the debris cleaning problems can bring insights for circular supply chain management under disruptions [115].

There is still a lack of scientific research that measures environmental and (indirect) social impacts of disruptions [101]. In the context of the global COVID-19 pandemic, it could be encouraged to obtain a set of economic and technological key performance indicators for vaccine supply chain design (see for example Lemmens et al. [116]). It would be helpful to capture a holistic view on supply chain performance indicators, to understand the implications of disruptions, prioritize mitigation actions and sustainably develop supply chain strategies, processes and capabilities for the future.

#### Stronger Consideration of Specifics of Context

The global COVID-19 pandemic is triggering multiple disruptions in the supply and distribution stages (including warehousing and transportation). Furthermore, it leads to increased demand fluctuations. Previous literature reviews already highlight the importance of distinguishing and combing the effects of these disruptions [31, 113, 117]. Different disasters tend to have drastically different characteristics. These aspects gain attention in the most recently published papers as well [34]. Aldrighetti et al. [93] suggest to take specifics from COVID-19 into consideration and look at it from a multi-period perspective [31, 97, 118]. It is encouraged to approach supply chain disruptions considering different phases such as response and recovery [80], proactive and reactive strategies [68], and incorporating different levels of preparedness [34] and resilience [58, 109].

Supply chain networks [89] and design [62, 118, 119] might affect implications and propagations of disruptions. It could be worthwhile considering supply chain structural aspects as moderator variables in future research studies. Additionally, other specifics such as industries [113], market position [55], size of the organization [113, 119], and location [119] can derive further insights on understanding supply chain disruptions in specific contexts and perhaps contribute to a more detailed understanding of the phenomenon.

Also for humanitarian researchers, contexts of the disruption and the impacted beneficiaries are relevant for further investigations, for example, distinguishing between characteristics of slow-onset versus sudden disasters [120]. Demands can shift over time and can be distributed unevenly between affected communities and people [69]. Constraints and service levels can change due to external factors (for instance external traffic and traffic diversions impacting evacuation plans [69].

Commercial and humanitarian supply chains can benefit from an exchange (see for example Holguín-Veras et al. [96]). There are learning opportunities for both in terms of strategy, tactical decision making and operational best practices pre, during and post disasters. Examples of successful cooperation also become visible during the ongoing COVID-19 pandemic for example with global distribution of vaccines [121]. Disaster management is characterized as a combination of command/control and improvisation (for example refer to Harrald [122]). This approach could be also applied and investigated in a commercial context. When disruptions require operational process and strategic changes, a potential question could be linked to temporary versus permanent supply chain setups. This concept is illustrated for example by Jahre et al. [123, 124]. Further thinking along this perspective, stakeholders might consider project and resource networks in the future to pool risks and opportunities to combat supply chain disruptions. At the same time, there might be a learning opportunity for humanitarian permanent supply chains, since there is a call for more preparedness activities in humanitarian supply chains [125, 126].

#### Stakeholder Cooperation, Interaction and Human Behavior

Behavioral research can significantly advance theory and practice in supply chain management [127]. In the context of investigating supply chain disruptions, various levels of behavioral studies [28, 61, 68, 72, 90, 95] are possible: individual, organizational and network relational aspects.

In this context, the individual managers’ perception of resilience and their risk personalities [77] can provide additional insights. Besides risk neutrality, different personal risk behavior attributes, such as risk aversion can be investigated [68, 87]. Organizational behavior regarding management of risks, disruptions, and decision making are under-explored areas in operations research and management science [88, 113].

Network relational aspects could bring insights to risk propagation [61] based on risk behavior of stakeholders [128]. This might affect decision making [28, 77], information flow [41], and information asymmetry [89] along the value chain. Perceptions of risk might also vary based on the role of the stakeholder [129, 130] within the supply chain. It could be investigated how changes in consumer behavior due to reduced risk perception could affect demand uncertainty [131]. Cooperative structures promote coordination and integration among supply chain partners (customers, suppliers and other organizations in the network) in critical activities [119]. Based on their function (for example in public–private partnerships), the distribution of risks varies among project participants [67] and value chain stakeholders. Within the humanitarian research context, the organization of involved parties [69], development of partnerships [132], and consideration of interdependencies between agencies [133] continue to feed research opportunities.

Additionally, behavioral risks have attracted less attention [46]. Incorporating the human factor into future extensions of the present research [55], also amid the COVID-19 pandemic, is an additional level of consideration during the disruption and how it impacts reactions and recovery progress.

#### Trade-offs (e.g., Between Resilience and Costs)

Trade-offs [134] describe alternatives that cannot be fully satisfied simultaneously. Common commercial supply chain challenges consider balancing distribution costs with shipment rates, or overall logistics costs and service levels [87]. Within supply chain disruption research multiple authors mention to consider trade-offs [38, 114, 135, 136]. Especially balancing investments into resilience [75], efficiency versus flexibility [135], activities, and costs [55, 75] related to potential risks and mitigation actions should be put in perspective.

#### Implications of Digitalization

Digitalization can be considered as a threat [46, 54, 111], mitigation action [45], or as a method in the context of supply chain disruption research [34, 55, 95, 106, 135]. Understanding the role of supply chain digitalization with innovative technologies [58] will highlight new opportunities to address supply chain disruptions. For instance: artificial intelligence [131], machine learning [54, 78], big data analytics [64, 72, 131, 135], block chain [45], and e-commerce. Examples for applications could be digital twins [55], virtual reality-based simulation [55], additive manufacturing [39, 135] and industry 4.0 [54, 72]. Additionally, especially humanitarian supply chain literature reviews address the use and role of social media to be better prepared for upcoming disasters [29].

#### Quantitative Methods: Longitudinal, Multi-method, Multiple Objectives

The application and details of different quantitative methods, to better describe and understand disruptions in supply chains, are mentioned in the research opportunities of the literature reviews. In general, there is a need for quantitative analysis [133, 137], solution methods [133], and simulation of the disruption phenomenon and uncertainty [62, 118].

The concepts of resilience [60, 62, 75], responsiveness [118], flexibility, trade-offs [135], and context [138], as mentioned before, can be incorporated into a quantitative analysis. It is necessary to define relevant supply chain objectives [69, 87]. With that, multi-objective models describe the circumstances in more dimensions than single objective analyses [58, 115]. This can integrate models that are based on real-world data [93] in real-time [28].

Additional elements might be worthwhile incorporating, such as risk [33, 97], recovery [88], disruption, and propagation processes [68, 137]. The development of proxy methods [139] and selecting (proxy) indicators [78] is proposed.

There are various suggestions by authors for different quantitative methods and techniques, for example: scenario development and sampling [118], Bayesian networks and Markov chain modeling [54], heuristic and metaheuristic [136], game theory [44], and input–output analysis [101]. Additionally, variational inequalities [140, 141], which are not extensively covered in the reviewed literature reviews, could be further applied. They have a rich history of providing insights with respect to supply chain network disruptions (e.g., [142, 143] and could provide a complementary perspective (e.g., [144, 145]).

Due to the nature of disruptions, there is an emphasis on longitudinal investigations over multi-periods [97, 118] and strategies over time [20, 55, 75]. In order to address the complexity and multiple elements of supply chain disruptions it seems encouraging to use multi-method [146] approaches and cross-disciplinary work [109], for example by combining simulation and empirical research [114].

#### Qualitative Methods

The topics described earlier can also be investigated from a qualitative perspective [74], for example resilience, mitigation actions, human and organizational behavior. There seems to be a lack of field studies [74, 90, 114], where the COVID-19 situation could serve as a vivid exploratory setting. It could also be used to explore strategies over time from a longitudinal [ 75, 90] perspective. The application of complexity theory [114], grounded theory [76], knowledge-based theory [76] and real-life case studies [117] can increase the understanding of disruption phenomena. The combination of methods [74, 75, 109, 114, 146], together with quantitative approaches, helps to compare and validate [61] the results. The additional insights can deepen the understanding of supply chain complexity [62], scenario development [72], mitigation capabilities [62], and decision making under uncertainty [76].

## Conclusion

A vast array of publications is considering supply chain disruptions in the context of commercial, humanitarian, and public supply chains. This chapter reviews 93 literature reviews, which were published between 2006 and June 2021. Key terms mentioned are risk, resilience, operations and humanitarian. The framework clusters supply chain disruption research based on the CIMO logic into context, source of disruption, implications on supply chain stages (such as supply, distribution, demand and propagation effects), method and supply chain performance.

The research opportunities identified in the literature reviews can be synthesized as follows: definition and characteristics of resilience, measuring the impact of disruptions on supply chains, considering sustainable supply chain performance indicators, taking specific contexts into account, including studies on human behavior and digitalization. The definition and measurement of resilience as well as the impact of disturbances on supply chain performance are of outmost importance. Moreover, the consideration of sustainability in the context of the triple-bottom line and circular supply chains finds attention. A stronger consideration of specific contexts of the disruption and the investigated supply chain node(s) can provide additional insights. Further research in the area of stakeholder cooperation, interaction and human behavior amid disturbances can identify opportunities and hurdles for organizations to cope with supply chain disruptions. Investigating trade-offs between different preparation and recovery activities, as well as their implications on costs might help find balanced decisions. Understanding the implications of digitalization as a threat, mitigation action and method can increase the understanding of its role for supply chains amid disruptions.

Various quantitative and qualitative research methods can be applied to study supply chain disruptions and how to cope with them. Especially real-time, multi-period and mixed-methods can be used to describe, explain and test implications of disruptions on supply chains. Particularly the on-going COVID-19 pandemic brings opportunities for researchers to gather data, test real-time implications of supply chain disruptions and empirically validate scientific theories.

This review has inherent limitations based on the study setup (selection of search-string) and time-frame set. Academic research was published after the dataset of this review was collected and is thus not considered in this paper. This meta-analysis is based on literature reviews. Primary articles and studies might have additional insights, which are not reflected in this paper. However, this chapter hopes to highlight and clarify elements of supply chain disruption research as a baseline for future research.

## Declarations

### Data Availability

Data will be made available on reasonable request.
